# Algor-ethics: charting the ethical path for AI in critical care

**DOI:** 10.1007/s10877-024-01157-y

**Published:** 2024-04-04

**Authors:** Jonathan Montomoli, Maria Maddalena Bitondo, Marco Cascella, Emanuele Rezoagli, Luca Romeo, Valentina Bellini, Federico Semeraro, Emiliano Gamberini, Emanuele Frontoni, Vanni Agnoletti, Mattia Altini, Paolo Benanti, Elena Giovanna Bignami

**Affiliations:** 1 Department of Anesthesia and Intensive Care, Infermi Hospital, Romagna Local Health Authority, Viale Settembrini 2, Rimini, 47923 Italy; 2 Health Services Research, Evaluation and Policy Unit, Romagna Local Health Authority, Viale Settembrini 2, Rimini, 47923 Italy; 3 Unit of Anesthesia and Pain Medicine, Department of Medicine, Surgery and Dentistry “Scuola Medica Salernitana, ” University of Salerno, Baronissi, Salerno, Italy; 4 School of Medicine and Surgery, University of Milano-Bicocca, Via Cadore, 48, Monza, 20900 Italy; 5 Dipartimento di Emergenza e Urgenza, Terapia intensiva e Semintensiva adulti e pediatrica, Fondazione IRCCS San Gerardo dei Tintori, Via Pergolesi, 33, Monza, 20900 Italy; 6 Department of Economics and Law, University of Macerata, Macerata, 62100 Italy; 7 Anesthesiology, Critical Care and Pain Medicine Division, Department of Medicine and Surgery, University of Parma, Via Gramsci 14, Parma, 43125 Italy; 8 Department of Anesthesia, Intensive Care and Prehospital Emergency, Ospedale Maggiore Carlo Alberto Pizzardi, Largo Bartolo Nigrisoli, 2, Bologna, 40133 Italy; 9 Department of Political Sciences, Communication and International Relations, University of Macerata, Macerata, 62100 Italy; 10 Department of Surgery and Trauma, Anesthesia and Intensive Care Unit, Maurizio Bufalini Hospital, Romagna Local Health Authority, Viale Giovanni Ghirotti, 286, Cesena, 47521 Italy; 11 Hospital Care Sector, Emilia-Romagna Region, Via Aldo Moro, 21, Bologna, 40127 Italy; 12 Pontifical Gregorian University, Piazza della Pilotta 4, Roma, 00187 Italy

**Keywords:** Algorethics, Artificial intelligence, Machine learning, Ethics, Data engineering

## Abstract

The integration of Clinical Decision Support Systems (CDSS) based on artificial intelligence (AI) in healthcare is groundbreaking evolution with enormous potential, but its development and ethical implementation, presents unique challenges, particularly in critical care, where physicians often deal with life-threating conditions requiring rapid actions and patients unable to participate in the decisional process. Moreover, development of AI-based CDSS is complex and should address different sources of bias, including data acquisition, health disparities, domain shifts during clinical use, and cognitive biases in decision-making. In this scenario algor-ethics is mandatory and emphasizes the integration of ‘Human-in-the-Loop’ and ‘Algorithmic Stewardship’ principles, and the benefits of advanced data engineering. The establishment of Clinical AI Departments (CAID) is necessary to lead AI innovation in healthcare, ensuring ethical integrity and human-centered development in this rapidly evolving field.

## Introduction

Artificial intelligence (AI) algorithms have steadily gained prominence in healthcare and are expecting to revolutionize the landscape of clinical decision support systems (CDSSs) [1–3]. However, in the field of critical care, despite the increasing number of developed AI-based algorithms, the vast majority remains within the testing and prototyping phase, lacking external validation and certification [4]. CDSSs harness AI to enhance patient outcomes and assist healthcare professionals in making informed diagnostic and therapeutic choices. Theoretically, the amalgamation of AI’s advanced capabilities with the acumen and skill of clinicians represents a transformative force in healthcare. However, an intrinsic complexity of critical care intensifies the challenges associated with implementing AI-based methods. Therefore, ensuring fairness and responsibility in AI implementation is crucial. Relying solely on “algorithmic fairness audits” may not guarantee the equitable development and deployment of such technology [5, 6]. Instead, they should be integrated within a comprehensive and structured approach to AI implementation in critical care settings [7].

In the rapidly evolving landscape of healthcare, the concept of Algorithmic Stewardship emphasizes the judicious management and integration of these technologies, advocating for a robust framework that addresses potential biases and ensures clinicians’ readiness [8]. This framework underscores the importance of a synergistic interplay between humans and algorithms throughout the machine learning process grouped under the umbrella term of Human-in-the-Loop (HITL) [9].

Questions regarding ethical use and implications of AI in healthcare become increasingly critical and an ‘algor-ethics’ emerges at the forefront. This novel ethical paradigm addresses the dynamic nature of AI encompassing the entire process of its development, from its nascent stages to its real-world deployment. By setting the ‘rules of the game’, algor-ethics establishes the bedrock upon which Algorithmic Stewardship and HITL are built, aligning AI systems with human values and societal norms, and fostering accountability, fairness, and transparency. In this manuscript, we aim to present our perspective about the principles at the foundation of the AI development in critical care, starting by identifying the peculiarities in this field, addressing the most relevant sources of bias, and suggesting a structured process.

### Peculiarities to account of AI deployment in critical care

**Fig. 1:**
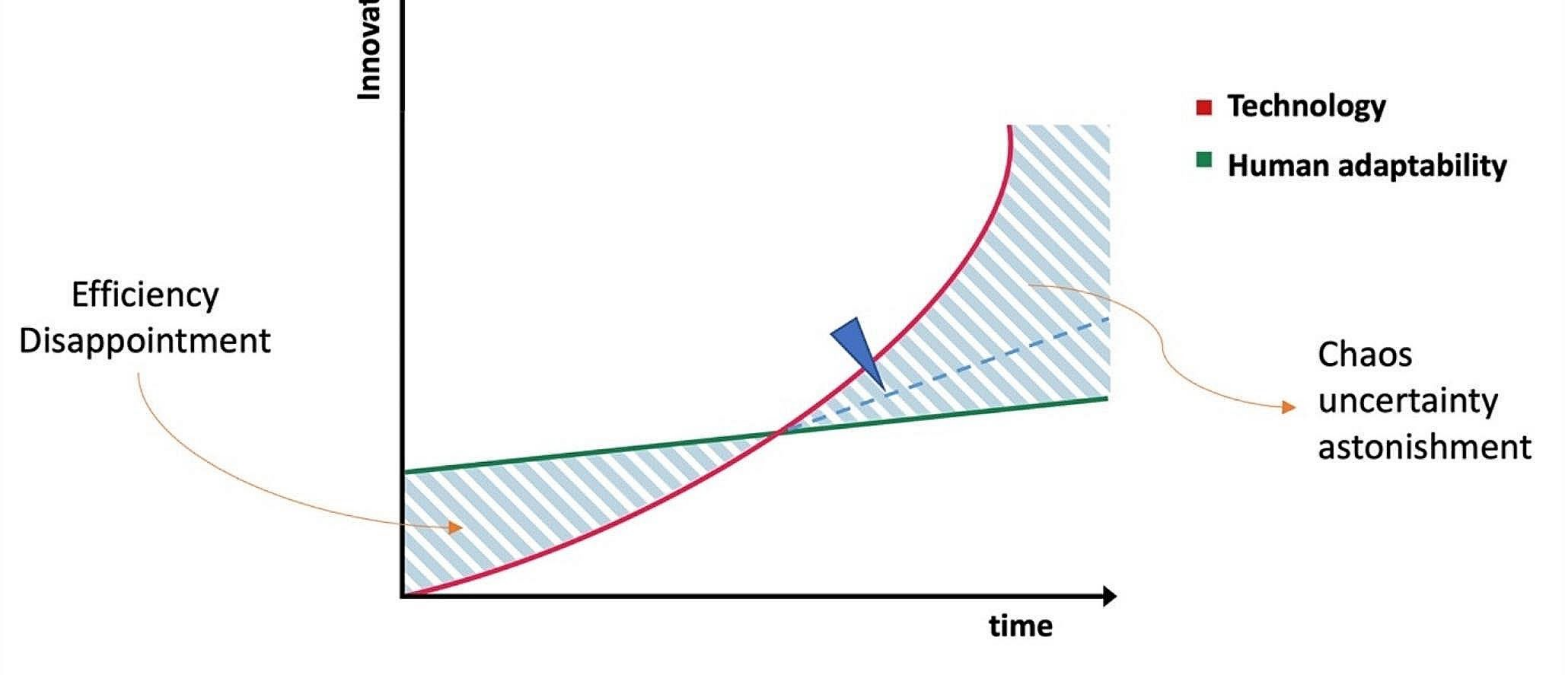
Graphical depiction illustrating the progression of technological innovation in relation to human adaptability. The evident divergence emphasizes the accelerated rate of technological advancements compared to human capacity for adaptation, leading to potential areas of disparity and unpredictability. Such observations necessitate a considered approach to technological developments to ensure alignment with human evolutionary pathways

Human beings have always actively promoted technological advancement as part of the human evolution. In the past, the rate of innovation was often limited by technology itself in comparison with human adaptability. However, recent significant advancements in the computational power, have exponentially accelerate technological growth to the point it has reach, or even outpaced, our ability to adapt and control such development (Fig. 1). This concept was firstly introduced by Thomas Friedman emphasizing the need for lifelong learning as a pathway to harmonizing our relationship with advancing technology [10]. Within healthcare, critical care heavily utilizes technology, ranging from continuous invasive and noninvasive monitoring [11], imaging tools [12, 13], and organ support devices such as ventilators [14, 15], and hemodialysis [16]. Consequently, a massive amount of data is continuously stored in electronic healthcare records (EHRs). This data could potentially serve to assist physicians in understanding the complexity and heterogeneity of patient conditions. However, data stored in EHRs are not designed for rapid assessment or to support timely decision-making. The fact that intensivist usually coordinates a team while being required to make quick decisions within limited information adds layers of complexity to decision-making. Unlike other specialties, the intensivist’s role often demands rapid decisions without direct patient input such as in the decision to withdraw life-sustaining treatment for futility or not to resuscitate [17]. These decisions are often precipitated by acute, unexpected events that preclude the possibility of discussing choices not only with the patient but sometimes even with their closest relatives. Therefore, the intensivist may face the profound responsibility of acting in the patient’s best interests based on clinical judgment and existing ethical frameworks, without the patient’s articulated consent or advanced directives. As we explore potentiality of AI in critical care, it is crucial to establish a deployment foundation that upholds the best interests of all individuals involved —patients, healthcare practitioners, and when possible, their families— while also confronting the inherent challenges this alignment presents.

### Bias in data acquisition for AI in healthcare

The accuracy and reliability of AI in healthcare are challenged by varied institutions’ practices. This variability affects AI performance across different settings. While data standardization is important, its broad application is impractical due to different clinical protocols and environments. Instead, the focus should shift towards the development of advanced data engineering such as Extract, Transform, Load (ETL) pipelines [18, 19]. These pipelines play a critical role in effectively harmonizing and integrating data from disparate sources and formats enabling the collection and processing of data in a manner that acknowledges the complexity inherent in critical care and respects the unique operational workflows of each healthcare institution [20, 21]. This approach allows the development of more robust and adaptable AI algorithms [22].

### Sources of bias in algorithm development for AI in healthcare

Although data engineering may markedly help with the collection and harmonization of data from different sources, health disparities can inadvertently be exacerbated by AI algorithms if not duly considered during the development and deployment stages [23]. “Algorithmic fairness audit” is a useful tool to prevent AI algorithms from unintentionally promoting health disparities that may stem from residual and unmeasured confounding, which may be associated with patients’ characteristics such as such as gender [24], comorbidities [25], or ethnicity [5]. Additionally, although the prognostic impact of comorbidities may be associated to ethnicity other associated conditions such as socioeconomic status and educational levels may partly explain the association and such information are not routinely recorded or available to researchers [26]. In such scenario, audits should evaluate the performance of the AI-based algorithms in population subgroups by different metrics and lead to an appropriate calibration and discrimination of the models. Nevertheless, health disparities are not solely a product of unmeasured or residual confounding. Historical biases present in training data, algorithmic biases favoring certain demographics, limited access to advanced healthcare technology, and cultural differences or language barriers, can all contribute to disparities [27]. Despite also other sources of bias have been reported in the phases of algorithm development such as the managing of missing data or outliers, features selection, or modeling of underrepresented groups [23, 28], data sharing restrictions and data privacy represent a fundamental issue that should be considered. Data protection and patient protection are both critically important in the context of healthcare. However, when implementing AI algorithms, it is necessary to question the priority between the two as they may, at times, conflict with each other or require different approaches for optimization. For instance, strict data protection may sometimes limit the availability of data needed for the development of AI algorithms that can potentially save lives or significantly improve patient outcomes. The dilemma is highlighted by Floridi, who has questioned the priority to data protection over patient protection [29]. While data protection is important, it should not be regarded as an absolute value, but rather as an instrumental value that serves broader ethical principles, such as the protection of human dignity and well-being. In some cases, the protection of patients may necessitate the use of data in a manner that can conflict with stringent data protection norms. If different approaches have been proposed to limit methodological sources of bias, in regard to data protection, the values and potential outcomes should be carefully weighted to arrive at decisions that uphold the core ethical principles of promoting well-being and protecting human dignity.

### Issues to take into consideration in the use of AI-based CDSS

In the AI deployment in healthcare it is vital to ensure that the advancements benefit all patient demographics equitably. Although the process of re-calibration and external validation may improve the generalizability of the algorithms, it poses the problem of the “domain shift” referring to the phenomenon wherein the statistical properties or distribution of the data that an algorithm is applied to during real-world usage differ from the properties or distribution of the data that the algorithm was trained on [30]. This discrepancy can be due to various factors including changes in population characteristics, technological advancements, evolving clinical practices, or even subtle differences in data collection methods between different settings. In the context of critical care, domain shift may significantly impact AI algorithm. For example, a predictive AI algorithm to diagnose acute kidney injury based on data where prevail data on prerenal causes might fail to accurately perform on patients developing intrarenal acute kidney injury. Domain adaptation techniques are often employed to tackle domain shift by adjusting the algorithm to perform better on the new data distribution without the need for extensive retraining (i.e. transfer learning, feature-level domain adaptation, instance-weighted domain adaptation) [31, 32]. In recent years, there have been also advancements in machine learning that specifically address fairness such as adversarial de-biasing, where a model is trained to make predictions that are statistically independent of certain sensitive attributes, such as gender or ethnicity [33]. Moreover, the introduction of AI-based CDSS is not merely an addition to the existing clinical practice but a transformative influence that reshapes it. As these systems are integrated into the workflow of critical care, where novel monitoring systems and experimental diagnostic tools are frequently adopted, the practice itself evolves. This evolution can alter the predictive accuracy of AI-based CDSS, necessitating their continuous adaptation to the shifting clinical landscape. Particularly in critical care, the rapid pace of technological innovation and the trial of emerging treatments demand that AI systems are not static but are capable of learning and adapting in tandem with the field’s advancement.

To sustain the relevance and accuracy of these systems, an ongoing process of audits and performance supervision is indispensable. This continuous oversight ensures that AI-based CDSS remains attuned to the ever-changing reality of patient care, capable of identifying domain shifts and deviations from established data patterns. By institutionalizing a regimen of rigorous, ongoing evaluation, healthcare providers can maintain the integrity and utility of AI applications, ensuring these tools continue to reflect and respond to the latest clinical evidence and practice standards.

### Human factors contributing to bias in the use of AI in healthcare

Human reasoning is not inherently structured for optimal decision-making but rather for maximizing decision-making efficiency. This understanding is crucial in healthcare, where the decisional process is usually complex and clinical judgment should follow a structured pathway and should not merely follow the intuitive approach [34]. The dual-process theory differentiates between ‘System 1’ and ‘System 2’ thinking: System 1 operates quickly and intuitively, often relying on heuristics, while System 2 is slower, more deliberate, and analytical [35]. Clinicians frequently rely on System 1 for immediate, routine decisions, but complex, high-stakes scenarios necessitate the engaged, analytical reasoning of System 2. Understanding this dichotomy is pivotal in grasping how healthcare professionals interact with AI-based systems and the potential biases that can arise from these interactions. In critical care, the urgency of decision-making often leads clinicians to predominantly utilize System 1 thinking. This reliance on intuition and heuristics, while efficient, can be prone to biases and errors. Recognizing, evaluating, and addressing these cognitive dynamics is crucial when considering the use of IA-based CDSS. Gaube et al. highlight a critical concern where physicians might unconsciously adopt biases present in AI systems [36]. Diagnostic accuracy was significantly worse when participants received inaccurate advice. Notably, the ability to detect the low quality of the advice from the AI system correlated with the degree of the physician task-expertise. This phenomenon underscores the risk of a ‘feedback loop’ where AI becomes a source of reinforced bias. Over-reliance on AI might lead clinicians to overlook its limitations, leading to potential errors in judgment when AI advice is flawed [37]. Nonetheless, the bias carried by an AI system affects clinicians’ decisions also when AI is no longer making recommendation suggesting human inheritance bias of AI [37]. The evidence collectively calls for a balanced approach to AI implementation in healthcare. It necessitates not only technical precision but also an awareness of human cognitive biases and the development of a digital culture among all medical stakeholders to critically engage with AI-based CDSS [38]. This approach is especially pivotal in critical care, where the ramifications of decision-making are immediate and profound.

### Algor-ethics: ensuring ethical integrity in AI development and implementation in healthcare

**Fig. 2:**
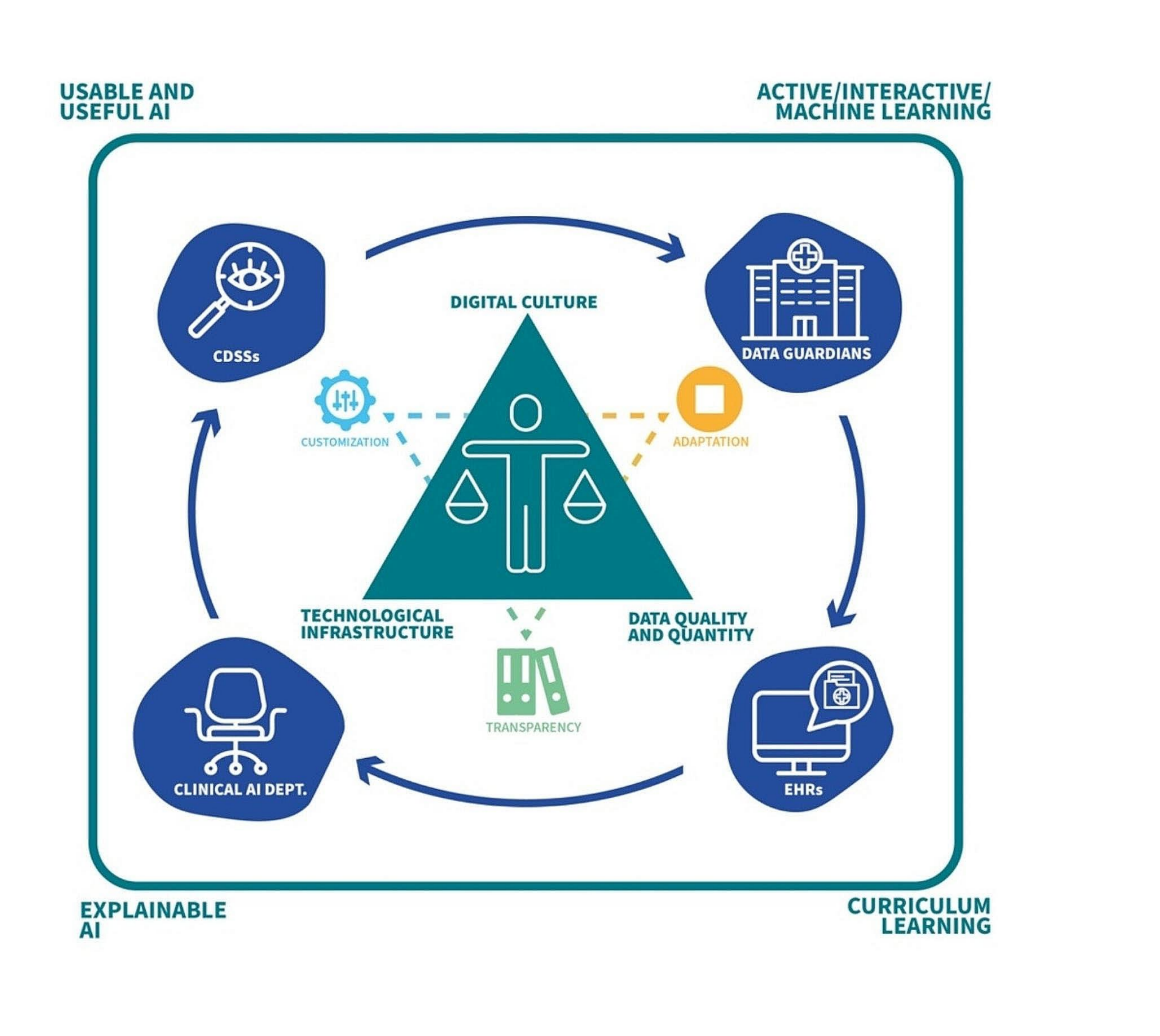
A Mind Map of AI Application in Healthcare. The looping structure illustrates the various steps in the development and deployment of AI models, starting with data acquisition from hospitals or institutions (data guardians), to the storage of this information in electronic health records (EHRs), to the prototyping and validation phase of AI algorithms within a clinical AI department (CAID). The process culminates in the implementation of clinical decision support systems (CDSSs) that can alter clinical practice. This cycle is built upon the three pillars of digital transformation: data quality and quantity, a sound technological infrastructure, and a nurturing digital culture. Algorithmic stewardship guides and oversees the various domains: learning with humans, curriculum learning, explainable AI, and Beyond learning – Useful and Usable AI. This entire process aligns with the principles of algor-ethics, putting humans at the center of the process and adhering to the principles of traceability, customization, and adequation. This underscores the human-centered approach of algor-ethics, with AI designed to augment human decision-making and adapt to the unique needs and interests of each individual. It serves as an ethical layer that embraces and interacts with all aspects of the AI development and deployment process

Prioritizing ethical considerations sets the stage for responsible and beneficial AI integration, reinforcing the commitment to patient welfare and equitable healthcare practices. Notably, the leadership of this process should originate from the identification of specific healthcare needs, aims, and priorities, ensuring that technology serves these predefined objectives. This human-led direction ensures that technological development is a response to actual clinical demands, rather than developing algorithms first and subsequently seeking applications for them. This approach helps to avoid the pitfall of technology-driven solutions in search of problems, focusing instead on problem-driven innovations that are more likely to benefit patient care and healthcare outcomes. To this end, it is crucial to devise an ethical framework that can address the complexity and value of humans - their unique rational and emotional functionalities, and the intrinsic relationship between humans and artifacts that underlies our existence (techno-human condition). The unique challenges in critical care further underscore the need for an ethically guided, human-centric approach to AI implementation. The ethical framework previously advocated should, therefore, not only addresses the technical and clinical aspects of patient care but also deeply considers patient autonomy and dignity. This framework is “algor-ethics” [39] and must be thought of in a cooperative manner. In other words, AI is not an evolutionary adversary but a tool that must be thought of as cooperative with the person. Indeed, AI must be created to augment the cognitive capability that is a unique and peculiar prerogative of the human being, and they must never replace it. The ultimate objective, then, is to enhance human cognition, not to turn cognition into an algorithmic function separated from human beings. In this regard, the definition of the “wayfinding” approach for the purpose of AI is particularly helpful [40]. A shift towards a “generalized wayfinding” approach both in the diagnostic and therapeutic process may significantly improve the entire care process by helping with the understanding of the pathophysiological mechanisms underlying the development of clinical complications [41]. Moreover, such an approach promotes a culture of continuous learning with the aim of improving the path to the correct diagnosis, synthesizing complex patient data, and determining the best next steps, rather than predicting a predefined outcome. AI’s growing autonomy and decision-making capability necessitate control mechanisms to ensure safety, ethical alignment, and accountability. Furthermore, the implementation of AI technologies carries a displacement of “power” that could impact who benefits from and who is adversely affected by these technologies. Without an ethical framework, the use of AI could lead to inequitable outcomes, potentially benefiting some while inadvertently harming others [28, 42]. The role of algor-ethics can be elucidated through an analogy between AI-based algorithms and cars. The manufacturing process of cars, akin to the development framework, ensures that cars are equipped with essential safety features like brakes and steering. However, the mere presence of safety features does not guarantee accident-free roads. It is imperative for constructors and drivers, akin to developers and users of AI algorithms, to adhere to a set of rules and behaviors to ensure safety. This analogy underscores the importance of not only having safety features (ethical considerations in development) embedded in AI algorithms but also ensuring that they are implemented and employed within a responsible and regulated behavioral framework. Algor-ethics also guides the technical and pathway aspects of algorithmic development. To achieve this comprehensive scope, algor-ethics embraces two pivotal concepts: ‘Human-in-the-Loop’ (HITL) and ‘Algorithmic Stewardship’ (Fig. 2).

The ‘Human-in-the-Loop’ (HITL) concept is critical to question the role of human expertise and judgment within the AI workflow and outlines four main domains that encapsulate the interplay between humans and algorithms [9]:

- “Learning with Humans”: It delineates who controls the learning process, distinguishing between active learning (algorithm-controlled), machine teaching (human-controlled), and interactive machine learning (a dynamic and cooperative interaction);
- “Curriculum Learning”: This refers to the algorithm’s iterative learning process, whereby simpler tasks precede more complex ones, mimicking the learning trajectory of a human learner;
- “Explainable AI”: Given that AI systems often operate as a ‘black box’, transparency is key. Explainable AI ensures that AI model decisions are interpretable and transparent, promoting trust among clinicians.
- “Beyond Learning – Useful and Usable AI”: AI models need to be both useful (improving outcomes or efficiency) and usable (seamlessly integrating into existing workflows) to successfully enhance clinical practice.

“Algorithmic stewardship” has been recently advocated and may help with defining a framework capable of addressing these four domains and creating an AI that can be effectively integrated and improve clinical practice [8]. Algorithmic Stewardship should provide guidelines on AI accountability, transparency, and fairness, enabling a balance between human judgement and automated decision-making. It oversees the entire lifecycle of AI systems: their training, validation, implementation, and ongoing evaluation. Crucially, it ensures that AI applications enhance patient care without compromising ethical standards. To be effective, the process to define the “algorithmic stewardship” should have a collaborative governance promoting the collaboration between various stakeholders, including technologists, ethicists, regulators, and the healthcare professionals.Furthermore, it should be locally adapted to the characteristics of each institutions in term of the maturity of the “three pillars” of digital transformational: data quality and quantity, technological infrastructures, and digital culture (Fig. 2) [43].

Although we commonly think that “two heads are better than one”, it is crucial to acknowledge that in human-AI interactions, this synergy is likely missing. Indeed, the effectiveness of such collaboration depends significantly on the task’s nature and uniformity of the knowledge between human and AI [44]. Given that AI-based models are trained on extensive data and undergo intricate data processing not easily interpretable, the interaction is prone to knowledge asymmetry between the parties [44]. In setting requiring rapid interventions such as critical care, this imbalance increases further worsening the interaction [45]. One potential consequence is facing the situation where technology overcome human expertise, transforming physicians in passive executors. Although consequences of the imbalance are difficult to predict there is almost no doubt that such scenario is likely to impair clinical reasoning and motivation while increasing dependence on technology [46]. Algor-ethics, emphasizing a human-centered approach, works to ensure a meaningful engagement of physicians and other operators with tasks and environments.

In conclusion, the main requirements of algor-ethics may be summarized as following (Fig. 2):

- *Human-centered*: AI should not replace, but rather augment human decision-making. AI should preserve uncertainty in its output, providing information about the accuracy and precision of its estimates. This allows for the preservation of human intervention in the decision-making process, rather than ceding complete control to AI. Similarly, the scope of the algorithm should be calibrated to serve the patient, addressing specific and meaningful problems maximizing the use of limited resources. This approach ensures that AI not only supports medical professionals but also contributes efficiently to patient-centered care.
- *Traceability*: every step of the algorithm’s journey, from creation to validation, must be transparent and documented. An AI’s applicability, its outputs, and its developmental steps should be disclosed in a standardized datasheet.
- *Customization*: instead of a one-size-fits-all approach, AI algorithms must be capable of adapting and interacting with each individuality. This adaptability should be evident from both the perspective of the users (healthcare professionals) and the patients themselves.
- *Adequation*: The ultimate aim of AI in healthcare should be to align with and serve the patient’s best interest. The priority of the patient should become the priority of the algorithm, not vice versa.

### The clinical AI department: the place for a human-oriented AI development

The development and exploitation of AI-based algorithms involves multifaceted considerations, from data acquisition and quality to awareness of algorithmic biases and training of healthcare professionals. These advancements have the potential to enable physicians to make more informed diagnostic and therapeutic decisions, emphasizing the necessity for a seamless and effective digital technology integration in healthcare. The Clinical AI Department (CAID) is envisioned as a pivotal entity for addressing the complexities surrounding AI-based CDSS in healthcare [47]. As a global virtual institution comprised of numerous local CAIDs, it embodies the principle of “think globally, act locally”. This initiative not only bolsters data-driven decision-making but also triggers a virtuous cycle of perpetual enhancements in medical diagnostics and therapeutic methodologies (Fig. 2). The CAID should lead the AI innovation in healthcare, serving as a collaborative hub where stakeholders converge to identify problems, define strategies, assess infrastructure and feasibility, recognize limitations, develop algorithms, and evaluate the use and performance of AI as CDSS. Furthermore, it actively fosters an AI-informed culture within healthcare. Local CAIDs are crucial in promoting an ETL framework and contributing to national and international projects, ensuring a cohesive and comprehensive approach to AI in healthcare. This collaborative approach not only enhances the algorithm’s reliability and accuracy but also ensures that the AI tools developed are equitable, contextually relevant, and capable of addressing the diverse needs of patient populations.

## Conclusions

Although the integration of AI into clinical practice may significantly enhance patient outcomes and support clinician in the decisional process, it comes with distinct challenges. There is a need of a strong leadership defining the aims and the pathway to follow for the AI revolution in healthcare. The CAID may carry thing challenge by promoting a collaborative and multidisciplinary environment while safeguarding against biases and ensuring equitable patient care This work underscores the importance of algor-ethics as the foundation for AI development in healthcare, balancing technological advancement with the preservation of human values, autonomy, and the complexities of clinical decision-making. The future of healthcare AI lies not just in technological innovation but -further - in its harmonious integration with human expertise and vision, ensuring that AI serves as a tool for timely enhancement rather than replacement, ultimately contributing to improved patient outcomes and healthcare delivery.

## Data Availability

Not applicable.
